# Intracranial Pressure–Derived Cerebrovascular Reactivity Indices, Chronological Age, and Biological Sex in Traumatic Brain Injury: A Scoping Review

**DOI:** 10.1089/neur.2021.0054

**Published:** 2022-01-25

**Authors:** Carleen Batson, Kevin Y. Stein, Alwyn Gomez, Amanjyot Singh Sainbhi, Logan Froese, Arsalan Alizadeh, Francois Mathieu, Frederick A. Zeiler

**Affiliations:** ^1^ Department of Human Anatomy and Cell Science, Rady Faculty of Health Sciences, University of Manitoba, Winnipeg, Manitoba, Canada.; ^2^ Section of Neurosurgery, Department of Surgery, Rady Faculty of Health Sciences, University of Manitoba, Winnipeg, Manitoba, Canada.; ^3^ Biomedical Engineering, Faculty of Engineering, University of Manitoba, Winnipeg, Manitoba, Canada.; ^4^ Interdepartmental Division of Critical Care, Department of Medicine, University of Toronto, Toronto, Ontario, Canada.; ^5^ Centre on Aging, University of Manitoba, Winnipeg, Manitoba, Canada.; ^6^ Division of Anaesthesia, Department of Medicine, Addenbrooke's Hospital, University of Cambridge, Cambridge, United Kingdom.

**Keywords:** age, autoregulation, biological sex, traumatic brain injury

## Abstract

To date, there has been limited literature exploring the association between age and sex with cerebrovascular reactivity (CVR) in moderate/severe traumatic brain injury (TBI). Given the known link between age, sex, and cerebrovascular function, knowledge of the impacts on continuously assessed CVR is critical for the development of future therapeutics. We conducted a scoping review of the literature for studies that had a direct statistical interrogation of the relationship between age, sex, and continuous intracranial pressure (ICP)-based indices of CVR in moderate/severe TBI. The ICP-based indices researched included: pressure reactivity index (PRx), pulse amplitude index (PAx), and RAC. MEDLINE, BIOSIS, EMBASE, SCOPUS, Global Health, and the Cochrane library were searched from inception to June 2021 for relevant articles. A total of 10 original studies fulfilled our inclusion criteria. Nine of the articles documented a correlation between advanced age and worse CVR, with eight using PRx (2192 total patients), three using PAx (978 total patients), and one using RAC (358 total patients), *p* < 0.05; *R* ranging from 0.17 to 0.495 for all indices across all studies. Three articles (1256 total patients) displayed a correlation between biological sex and PRx, with females trending towards higher PRx values (*p* < 0.05) in the limited available literature. However, no literature exists comparing PAx or RAC with biological sex. Findings showed that aging was associated with impaired CVR. We observed a trend between female sex and worse PRx values, but the literature was limited and statistical significance was borderline. The identified studies were few in number, carried significant population heterogeneity, and utilized grand averaging of large epochs of physiology during statistical comparisons with age and biological sex. Because of the heterogeneous nature of TBI populations and limited focus on the effects of age and sex on outcomes in TBI, it is challenging to highlight the differences between the indices and patient age groups and sex. The largest study showing an association between PRx and age was done by Zeiler and colleagues, where 165 patients were studied noting that patients with a mean PRx value above zero had a mean age above 51.4 years versus a mean age of 41.4 years for those with a mean PRx value below zero (*p* = 0.0007). The largest study showing an association between PRx and sex was done by Czosnyka and colleagues, where 469 patients were studied noting that for patients <50 years of age, PRx was worse in females (0.11 ± 0.047) compared to males (0.044 ± 0.031), *p* < 0.05. The findings from these 10 studies provide preliminary data, but are insufficient to definitively characterize the impact of age and sex on CVR in moderate/severe TBI. Future work in the field should focus on the impact of age and sex on multi-modal cerebral physiological monitoring.

## Introduction

Cerebrovascular reactivity (CVR) is a surrogate measure of cerebral autoregulation: the change in blood vessel tone in response to changes in systemic blood pressure in an attempt to maintain constant cerebral blood flow (CBF).^1,2^ In traumatic brain injury (TBI), arteries often fail to respond appropriately to pressure changes, leaving the patient at risk for decreased CBF and ischemia or increased CBF and hyperemia.^3–6^ The literature suggests that patients with impaired CVR are more likely to suffer worse 6-month functional outcome.^7–10^

The three continuously updating intracranial pressure (ICP)-derived metrics—namely, pressure reactivity index (PRx), pulse amplitude index (PAx), and RAC (correlation [*R*] between slow waves of pulse amplitude of ICP [AMP] and cerebral perfusion pressure [CPP]) that received pre-clinical validation in experimental models—will be the focus of this scoping review. They are the most commonly described metrics of continuous CVR measurement in adult moderate/severe TBI.^3,7,9,11–13^ PRx is the most prominent in the literature.^3,13^ It is derived from the moving Pearson's correlation coefficient between slow waves of ICP and mean arterial pressure (MAP).^13,14^ Positive PRx values represent disturbed autoregulation whereas negative values represent preserved autoregulation.^7^ PAx is derived from the moving Pearson's correlation coefficient between slow waves of AMP and MAP.^7,13,15^ This index appears to outperform PRx at identifying impaired CVR in the setting of reduced ICP.^15^

The final ICP-based cerebrovascular index is RAC, derived from the moving Pearson's correlation coefficient between slow waves of AMP and CPP.^7,16^ This index behaves similar to PRx regarding mortality and outcome at 6 months post-TBI in some studies^7,9,11^; however, more work is needed with this index given that its input in the field is considered experimental at the moment.^16^ Data on PAx and RAC are more limited, but it is important for research into these metrics to continue given that they both provide useful CVR-based information and, additionally, compensatory reserve (measure of brain compliance post-injury)^17^ information in the case of RAC.^18^ PRx and PAx have received validation against the lower limit of autoregulation (LLA) for intracranial hypertension, and all three indices received validation against the LLA for pure arterial hypotension.^19–21^

A major challenge is that the literature supports no impact of current brain trauma foundation (BTF) treatments on CVR indices in moderate/severe TBI.^4^ This is exemplified by recent literature suggesting that, over the past 25 years of improvements in guideline-based care in moderate/severe TBI, there has been little impact on CVR measures and overall morbidity/mortality.^22,23^ Multi-center prospective high-frequency cerebral physiological data sets have confirmed treatment independence of CVR metrics,^24–28^ with upwards of 50–60% of any given day during the acute phase of intensive care unit (ICU) stay spent in a dysautoregulated state.^24,29,30^ As such, further understanding of the drivers of impaired CVR are critical to uncovering novel precision therapeutics directed at its prevention and treatment.

The first phase in uncovering drivers of impaired CVR involves comprehensive phenotyping.^31^ Work to date assessing the association between patient admission demographics and injury patterns with impaired CVR has been limited. During these preliminary assessments, age and biological sex have emerged as potential modifiers of cerebrovascular response after moderate/severe TBI.^32,33^ Age and biological sex are two key factors to be considered in the management of TBI. From the literature, it is seen that older patients tend to suffer worse outcomes from impaired CVR.^11,32,34^ This is because of aging processes and accumulation of amyloid-based substances, chronic inflammation, and compromised neuronal repair mechanisms.^35–38^ Biological sex is an important aspect to consider when discussing outcomes in TBI. It impacts cerebrovascular responses both independent of, but also potentially mixed-effects with, advancing age. It is understood that females are protected from inflammation and benefit from the protective vasodilatory effects of estrogen and even progesterone^39^ during their younger reproductive years; however, they lose these in their older years.^40,41^ It is well documented that circulating estrogen levels have an impact on cerebral vasculature, whereby it promotes the release of prostaglandin I2 (PGI2), a cerebral vasodilator, and, conversely, we observe in low estrogen states, as is the case in males (who dominate TBI cohorts),^32,42^ thromboxane A2 (TXA2) is dominant, which causes cerebral vasoconstriction and accompanying effects.^40,41^ It is evident from pre-clinical TBI models that progesterone has neuroprotective effects, with exogenous supplementation in animals resulting in reduced neuronal loss and improved neurophysiological and functional outcomes.^43,44^ Studies on this topic are limited, but will be a focus for future works.

Understanding the importance of having age and sex data in relation to CVR and its impact on outcomes is crucial in the management of TBI. This will help clinicians understand which patients are more likely to have a poor clinical course, which patients may potentially need more aggressive care, and various other outcomes can be anticipated, etc. The literature is scattered and scarce in this regard. Thus, in order to better understand the current knowledge gap in this area, we performed a scoping review of the literature for studies highlighting the association between age and biological sex with ICP-derived CVR indices in moderate/severe TBI.

## Methods

A scoping review was conducted using a systematic search of the literature in accordance with the Cochrane Handbook for Systematic Reviews.^45^ Data were reported by following the Preferred Reporting Items for Systematic Reviews and Meta-Analyses (PRISMA)^46^ and PRISMA Scoping Review (PRISMA-ScR) guidelines,^47^ with the PRISMA checklist shown in Appendix S A of the Supplementary Material.

### Search question, population, inclusion and exclusion criteria

The questions posed for this scoping review were: (1) What is the association between age and ICP-derived CVR indices in moderate/severe TBI?; (2) What is the association between biological sex and ICP-derived CVR indices in moderate/severe TBI?

Age was defined as any documentation of chronological age (in days, months, or years), whereas biological sex was defined as the binary designation (male or female). The three ICP-derived indices of interest were: pressure reactivity index (PRx; correlation between slow-waves of ICP and MAP)^14^; pulse amplitude index (PAx; correlation between slow-waves of AMP and MAP)^15^; and RAC (correlation [R] between slow-waves of AMP [A] and CPP [C]).^16^ These indices were chosen because they are the most readily discussed continuous ICP-derived CVR indices in moderate/severe TBI,^28–31^ have some degree of pre-clinical validation as measures of the autoregulatory curve,^19–21,48^ and have all demonstrated strong associations with long-term outcome in TBI care.^7,9,11–13,49^

### General inclusion/exclusion criteria

Inclusion criteria for this study were: studies with human subjects who suffered moderate/severe TBI (Glasgow Coma Scale [GCS] ≤12); patients 16 years and older (focusing on the adult cohort only); cohorts of ≥40 patients^50^; and published original works and studies with an objective direct statistical comparison of the ICP-based indices with age and biological sex. The cohort size cutoff of 40 was selected, given previous literature on sample size for cerebral physiological work in TBI indicating that this represents the absolute minimum for exploratory work.

Exclusion criteria for this study were: studies with patients who suffered mild TBI (GCS 13–15) or had no TBI; animal studies; patients <16 years of age; cohorts of <40 patients; non-original articles, non-English studies, non-ICP studies, non-standard/non-validated ICP indices^49^ (i.e., wavelet PRx [wPRx] or PRx variant [PRx_55-15_]); low-resolution ICP-derived indices (i.e., low-frequency autoregulation index [LAx] or long pressure reactivity index [L-PRx]); and studies with ICP indices that do not document the association with age and/or biological sex.

### Search strategy

To gather the necessary articles, MEDLINE, BIOSIS, EMBASE, SCOPUS, Global Health, and the Cochrane library were searched from inception to June 2021 using relevant search variables, which can be found in Appendix SB of the Supplementary Material.

### Study selection

All articles gathered by the database searches were downloaded into Zotero after deduplication. Two independent reviewers (C.B. and K.S.) conducted the initial screening of titles and abstracts to select articles that fit the inclusion criteria. A full-text review of the selected articles was then conducted by the same reviewers for the articles that had the associations of interest to answer our research questions. Discrepancies between the two reviewers were resolved by a third reviewer (F.A.Z.).

### Data collection

Data were extracted from the final selected articles and stored in an electronic database. Data fields included authors; country of study; age; sex; GCS Score; Marshall computed tomography Grade; pupillary response; number with traumatic subarachnoid hemorrhage (tSAH), epidural hematoma (EDH), subdural hematoma (SDH); primary objective of study; sample size; measure correlated with age; and measure correlated with sex and findings. These data can be found in Tables 1–3.

**Table 1.** Overview of Included Studies

Source (authors)	Country	Sample size (TBI patients)	Age	Sex (% male)	GCS score	Marshall CT grade	Pupillary response	No. with tSAH, EDH, SDH	The primary objective of the study
Czosnyka et al. (2005)^32^	UK	358 (only 158 used in analysis of age and PRx)	Range = 16–87	80% (288/358)	Range = 3–15 (20%, >8)				To ascertain whether cerebrovascular dysfunction impacts the relationship between age and outcome in patients post-TBI
Liu et al. (2017)^49^	UK	515	Mean = 38.4 SD = 16	75% (385/515)	Median = 7 IQR = 3–9				To compare the performance of transform-based wPRx with the traditional PRx
Steiner et al. (2002)^42^	UK	114	Range = 14–77 Mean = 34 SD = 16	84% (96/114)	Range = 3–14 Mean = 6.6 SD = 2.8			tSAH = 19 (17%) EDH = 14 (12%) SDH = 24 (21%)	To describe optimal cerebral perfusion pressure (CPP_OPT_) through constant monitoring of cerebral pressure reactivity in individual TBI patients
Aries et al. (2012)^15^	UK	327	Range = 15–87 Median = 36	75% (246/327)	Range = 3–15 Median = 6 (25%> 8)				To investigate the association between PAx and PRx in TBI patients utilizing long-term monitoring
Zeiler et al. (2020)^18^	Various centers in the EU	165	Median = 49 IQR = 29–64	78% (129/165)	Median = 7 IQR = 3–10	Median = 3 IQR = 2–6	BR = 125 (76%) UU = 15 (9%) BU = 25 (15%)	tSAH = 137 (83%) EDH = 41 (25%) SDH = 101 (61%)	To investigate admission CT markers of diffuse intracranial injury and their correlation with poor CVR in a multi-center cohort (CENTER-TBI)
Czosnyka et al. (2008)^33^	UK	469	Mean = 33	79% (371/469)	Median = 6				To explore the effect of sex on ICP, CPP, PRx, and outcome post-TBI
Zeiler et al. (2018)^34^	UK	358	Mean = 40.6 SD = 17.2	76% (272/358)	Median = 7 IQR = 3-9			tSAH = 273 (76%) EDH = 26 (7%) SDH = 123 (34%)	To investigate the association between intracranial, injury burden, extracranial injury burden, and abnormal physiology and their effects on CVR in TBI patients
Czosnyka et al. (2006)^50^	UK	429	Mean = 34 SD = 16.7	79% (339/429)	Range = 3-15 Median = 6 (20%> 8)				To examine the connection among longstanding computer-based monitoring of ICP and its related indices against the outcome, age, and biological sex
Hiler et al. (2006)^51^	UK	126	Range = 14–74 Mean = 38.5		Range = 3-14 (24%> 8)	Median = 2 IQR = 2–5			To ascertain the importance of preliminary CT scan results, ICP measures, and autoregulatory status in prognostication during the initial 24 h post-TBI
Radolovich et al. (2011)^52^	UK	293	Mean = 37 SD = 16		Median = 6				To correlate PAx (new index) and Mx as CVR measures post-TBI

BR, bilaterally reactive; BU, bilaterally unreactive; CENTER-TBI, Collaborative European NeuroTrauma Effectiveness Research In Traumatic Brain Injury; CPP, cerebrovascular perfusion pressure; CPP_OPT_, optimal cerebral perfusion pressure; CT, computed tomography; EDH, epidural hematoma; EU, European Union; GCS, Glasgow Coma Scale; ICP, intracranial pressure; IQR, interquartile range; Mx, mean flow index (Pearson's correlation between CPP and cerebral blood flow velocity [CBFV]); PAx, pulse-amplitude index (Pearson's correlation between arterial blood pressure [ABP] and pulse amplitude of ICP [AMP]); PRx, pressure reactivity index (Pearson's correlation between ICP and ABP); SD, standard deviation; SDH, subdural hematoma; TBI, traumatic brain injury; tSAH, traumatic subarachnoid hemorrhage; UK, United Kingdom; UU, unilateral unreactive; wPRx, wavelet pressure reactivity index.

**Table 2.** Findings Regarding an Association between ICP-Derived Continuous Cerebrovascular Reactivity Measures and Age

Source (authors)	Sample size (TBI patients)	Measure(s) correlated with age	Findings
Czosnyka et al. (2005)^32^	348 (only 158 used in analysis of age and PRx)	PRx	- There was a significant correlation between PRx and age (*R* = 0.24, *p* = 0.003), indicating a deterioration in cerebrovascular control with advanced age. - Multiple comparisons were not corrected for.
Liu et al. (2017)^49^	515	PRx	- There was a significant correlation between PRx and age (*R* = 0.24, *p* < 0.001), indicating a deterioration in cerebrovascular control with advanced age. - Multiple comparisons were not corrected for.
Steiner et al. (2002)^42^	114	PRx	- There was a significant correlation between PRx and age (*R* = 0.358, *p* = 0.0001), indicating a deterioration in cerebrovascular control with advanced age. - Multiple comparisons were not corrected for.
Aries et al. (2012)^15^	327	PRx and PAx	- There was a significant correlation between PRx and age (*R* = 0.17, *p* = 0.004) as well as PAx and age (*R* = 0.35, *p* < 0.001), indicating a deterioration in cerebrovascular control with advanced age. - Multiple comparisons were not corrected for.
Zeiler et al. (2020)^18^	165	PRx	- Mean PRx values above the threshold were associated with advanced age (for a threshold of 0, mean age above = 51.4 years vs. mean age below = 41.4 years; *p* = 0.0007). - Multiple comparisons were corrected for using Bonferroni's method.
Zeiler et al. (2018)^34^	358	PRx, PAx, and RAC	- Age was associated with PRx (*R* = 0.235, *p* < 0.05), PAx (*R* = 0.495, *p* < 0.05), and RAC (*R* = 0.390, *p* < 0.05). Advanced age was also associated with increased percentage of time spent above index thresholds. - Multiple comparisons were corrected for using Bonferroni's method.
Czosnyka et al. (2006)^50^	429	PRx	- There was a significant correlation between PRx and age (*R* = 0.24, *p* = 0.003), indicating a deterioration in cerebrovascular control with advanced age. - Multiple comparisons were not corrected for.
Hiler et al. (2006)^51^	126	PRx_24_	- Patients with disturbed pressure reactivity had a significantly greater mean age than those with intact pressure reactivity (patients with PRx_24_ > 0, mean age = 44 years; patients with PRx_24_ < 0, mean age = 33 years; *p* = 0.0004). - Multiple comparisons were not corrected for.
Radolovich et al. (2011)^52^	293	PAx	- There was a significant correlation between PAx and age (*R* = 0.424, *p* < 0.05), indicating a deterioration in cerebrovascular control with advanced age. - Multiple comparisons were not corrected for.

PAx, pulse-amplitude index (Pearson's correlation between arterial blood pressure [ABP] and pulse amplitude of ICP [AMP]); PRx, pressure reactivity index (Pearson's correlation between ICP and ABP); PRx_24_, pressure reactivity index for the first 24 h of monitoring; RAC, correlation between pulse amplitude of ICP (AMP) and cerebral perfusion pressure (CPP).

**Table 3.** Findings Regarding an Association between ICP-Derived Continuous Cerebrovascular Reactivity Measures and Sex

Source (authors)	Sample size (TBI patients)	Measure(s) correlated with sex	Findings
Czosnyka et al. (2008)^33^	469	PRx	- PRx was found to be worse in females than in males in the age group <50 years (males, 0.044 ± 0.031; females, 0.11 ± 0.047; *p* < 0.05), no such change was noted in patients >50 years. - Multiple comparisons were not corrected for.
Zeiler et al. (2018)^34^	358	PRx	- Sex was associated with impaired CVR in patients in the category PRx >0.25, *p* = 0.040. - Multiple comparisons were corrected for using Bonferroni's method.
Czosnyka et al. (2006)^50^	429	PRx	- PRx was found to be worse in females than in males (males, 0.04; females, 0.1; *p* = 0.022). - Multiple comparisons were not corrected for.

ABP, arterial blood pressure; AUC, area under the receiver operating curve; ICP, intracranial pressure; PRx, pressure reactivity index (Pearson's correlation between ICP and ABP); TBI, traumatic brain injury.

### Statistical analysis

Given that this was a scoping review of the impact of age and biological sex on continuously monitoring ICP-based CVR in moderate/severe TBI, and significant heterogeneity between studies existed, no formal meta-analysis was conducted.

### Bias assessment

Given that this was a scoping overview of the available literature, a formal bias assessment was not conducted.

## Results

### Search strategy results

**FIG. 1.**
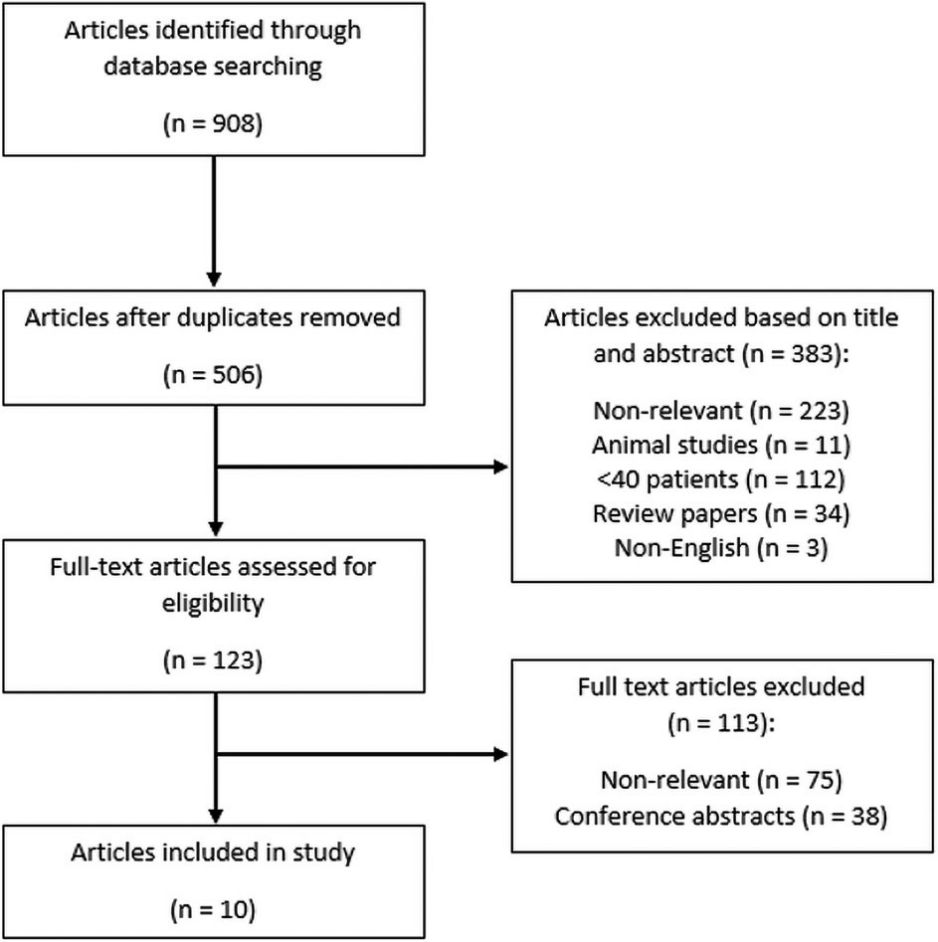
PRISMA flow diagram. PRISMA, preferred reporting items for systematic reviews and meta-analyses.

Figure 1 provides the PRISMA flow diagram detailing the search results. The database search returned a total of 908 articles. After deduplication, 402 articles were removed, leaving 506 for review. Article titles, abstracts, and keywords were initially screened in accordance with our inclusion/exclusion criteria, which resulted in 123 articles selected for full-text review. After the second screening of the full manuscripts, only 10 fully published original articles met the inclusion criteria and were used in this scoping review. All of these studies were conducted in the United Kingdom, except for one which was done from various centers in the European Union (EU). Nine of these studies were retrospective^15,32–34,42,51–54^ whereas one was prospective in nature.^18^ Table 1 presents an overview of the included study details.

### Indices and their statistical correlation to age

As seen in Table 2, an association between ICP-derived indices and age was made in nine of the ten selected studies. Eight of these studies referred to PRx, three to PAx, and one to RAC. Generally, it was noted that there was a significant correlation between advanced age and PRx, PAx, and RAC. We will highlight three of the most robust examples involving PRx, the three involving PAx, and the one involving RAC. Liu and colleagues compared PRx to age using a multi-variable binary logistic regression model of 515 patients (385 males, 130 females; mean age, 38.4 years; standard deviation [SD], 16), showing a relationship of *R* = 0.24, *p* < 0.001.^51^ Czosnyka and colleagues did their comparison using the correlation of 429 patients (339 males, 90 females; mean age, 34 years; SD, 16.7), expressing the relationship as *R* = 0.24, *p* = 0.003.^52^ Further, the final example by Aries and colleagues involved the use of the Spearman partial rank correlation of 327 patients (246 males, 81 females; median age, 36 years; range, 15–87) to represent the relationship between PRx and age, which was found to be *R* = 0.17, *p* = 0.004.^15^ PAx was compared to age by Aries and colleagues using the Spearman partial rank correlation of 327 patients (246 males, 81 females; median age, 36 years; range, 15–87), which showed the following relationship: *R* = 0.35, *p* < 0.001.^15^

The next study, done by Zeiler and colleagues, used Spearman's correlation matrix to compare PAx and age of 358 patients (272 males, 86 females; mean age, 40.6 years; SD, 17.2), showing a relationship where *R* = 0.495, *p* < 0.05.^34^ Radolovich and colleagues used *t*-tests and analysis of variance to analyze the relationship between these two variables of 293 patients (mean age, 37 years; SD, 16), resulting in *R*^2^ = 0.18, *p* < 0.05.^54^ The last highlighted study, done by Zeiler and colleagues, used Spearman's correlation matrix to compare RAC and age of 358 patients (272 males, 86 females; mean age, 40.6 years; SD, 17.2), showing a relationship where *R* = 0.390, *p* < 0.05.^34^ All mentioned studies displayed a weak-to-moderate positive correlation between age and worse CVR, as measured through PRx, PAx, or RAC.

### Indices and their statistical correlation to biological sex

As seen in Table 3, the only ICP-based index that was found to be correlated with biological sex was PRx in three of the ten selected studies. None of the other indices were correlated with sex in any of the included articles. The 2008 study by Czosnyka and colleagues of 469 patients (371 males, 98 females; mean age, 33 years) showed that for patients <50 years, PRx was found to be worse in females compared to males (females, 0.110 ± 0.047; males, 0.044 ± 0.031; *p* < 0.05).^33^ The study by Zeiler and colleagues of 358 patients (272 males, 86 females; mean age, 40.6 years SD, 17.2) showed that sex was associated with impaired CVR for those in the category PRx >0.25, *p* = 0.040, using univariate logistic regression.^34^ The final study, by Czosnyka and colleagues (2006), of 429 patients (339 males, 90 females; mean age, 34 years; SD, 16.7) demonstrated that females had worse mean PRx (females 0.1 vs. males 0.04; *p* = 0.022).^52^ However, from these results, it can be seen that the *p* values barely reached the threshold for statistical significance.

## Discussion

With this study, our group set out to gather literature to understand the relationship of age and sex with PRx, PAx, and RAC in TBI. From these 10 studies, we observed that worsening CVR is evident with increasing age.^32,33,42,51^ Females also appear susceptible to developing greater impairment in CVR in TBI. ^32,33,52^ The age-related findings are supported by the existing TBI and non-TBI literature on age and cerebrovascular function;^32,51,55–57^ however, the sex-related findings are unclear, with limited literature to date in moderate/severe TBI.

With regard to chronological age, the existing published literature demonstrates the effects of aging on the cerebral vasculature, whereby they lose their elasticity and overall integrity to function optimally.^32,36,55,57^ Also, collective vascular comorbidity (i.e., hypertension, diabetes, etc.) could be a causative factor in the gradual progression of autoregulatory dysfunction of the aged vasculature.^24,29,32^ It then should follow that in moderate/severe TBI populations, where continuous and validated measures of CVR are available, such age-related deterioration in cerebrovascular function should be apparent. This was the case, as reflected in nine studies where there was a statistically significant relationship (*p* < 0.05), but a weak positive correlation, between the ICP-based indices and increasing age, indicating that CVR worsens with advancing age.^32,33,42,51^

There were more studies involving PRx given that it is validated and more widely published^7,20,21,32,42,58^ with regard to TBI care. PAx and RAC displayed similar trends to PRx, with advancing age linked to worse PAx and RAC values, though the available literature was scarce, limiting the strength of conclusions that can be made regarding these two indices.^11,12,15,34^ PAx carries some degree of interest as an emerging CVR measure in moderate/severe TBI, given that in situations where ICP is persistently low (<15 mm Hg), it tends to be a better measure of impaired CVR, compared to PRx, as noted in studies by Aries and colleagues.^15^ On the other hand, RAC is still emerging in the literature, with current use relegated to an exploratory capacity.^7,16,34^

Biological sex displayed much less robust associations in the available literature identified. From the published biological sex-related literature, we understand that females have an advantage over males during their reproductive years because of the beneficial anti-inflammatory and vasodilatory effects of the key female sex hormones.^39–41^ From the 10 studies examined in this review, eight had sex data and it can be observed that males dominated the patient cohorts because the percentage of males ranged from 75% to 84%.^42,51^ However, only three studies were identified that made reference to sex and PRx^33,34,52^ whereas none made reference to PAx or RAC. Zeiler and colleagues showed that sex was associated with impaired CVR in the category PRx >0.25 where *p* = 0.040.^34^ The two studies by Czosnyka and colleagues showed a statistically significant relationship between PRx and females (*p* < 0.05).^33,52^ Their 2006 study showed that PRx was worse in females who were relatively young given that mean age was 34 years (SD, 16.7),^52^ with their 2008 study demonstrating that females <50 years of age had worse PRx than males of the same age category.^33^

These findings are surprising and contrary to what is expected biologically, but they are in keeping with similar findings in studies by Czosnyka and colleagues^33^ and Farin and colleagues,^59^ where we observed that clinically results could be conflicting and that young females are more prone to brain swelling, which can offset their hormonal advantages over males.^33,59^ Important to note is that in the 2008 Czosnyka and colleagues study,^33^ patients were separated by the age group <50 and >50 years, but the menopausal state of the women was not checked, which could pose some challenges.^33,40,41,59^ One cautionary note regarding these three studies is that the overall strength of statistical significance was limited (i.e., *p* values of <0.05, 0.040, and 0.022), with heterogeneous populations, grand averaging of long epochs of recorded physiological data, and no adjustment for multiple comparisons.^33,34,52^

### Limitations

Thus far, it can be seen that some promising findings are emerging in the literature regarding the relationship between age, biological sex, and the ICP-based indices on moderate/severe TBI. However, we must highlight some challenges that prevent conclusive findings. To begin, very few studies were found meeting our criteria to address such a critical topic. Of these few studies, even fewer addressed the sex aspect of our study. Also, studies contained a small number of females (which is usually the case in the TBI literature) whose menstrual/menopausal status has not been ascertained to garner the necessary information and the information was conflicting at best. Studies containing predominantly males can give a false notion that males are suffering worse outcomes. Small numbers affect the power of the study.

Of note, all studies mainly included gross averaging of data to obtain results.^32,34,51^ Insult burden was not looked at in any great detail to ascertain the relationship between different thresholds of the indices and how various patients are affected based on their age group or sex designation.

Next, patient populations from these studies were very heterogeneous with regard to demographics, injury pattern, duration of monitoring, and variance in received therapies between patients and centers, which may create confounding bias. All patients have different backgrounds, comorbidities, and injury patterns.^15^ Also, ICU therapies will vary based on their particular level of trauma, complications, and systemic issues. Some of the treatments (e.g., ventilation, temperature changes, medication, etc.) can affect vascular dynamics, physiology variables, and cause interference in data recording.^15^

Fourth, overall study sizes were small compared to other TBI studies without high-frequency physiology.^60^ Hence, the strength of the conclusion regarding age, sex, and CVR is limited. Furthermore, many studies evaluated various patient demographic features with CVR without correcting for multiple comparisons. As such, the statistically significant findings should be taken with caution and as preliminary findings only. We elected to use the cut-off value of 40 for sample size based on previous literature defining this as the minimum number for exploratory work in cerebral physiological studies in TBI.^50^ It must be acknowledged that this previous work also found that the optimal sample size was 100 patients or higher for such studies if one wanted to make more definitive comments on associations observed. Our selection of 40 for this review was based on our desire to be all-inclusive. However, in doing so, studies with small cohorts between 40 and 100 patients will admittedly not necessarily have over 40 female patients, given the differences in male versus female representation in TBI incidence. Thus, the strength of conclusions for those included manuscripts with cohort sizes <100 are limited at best.

Fifth, only studies where CVR was assessed by ICP-derived measures were analyzed. This was the case because they have the most pre-clinical validation and widespread applicability to TBI care/monitoring, therefore being most suited for this review on the impact of aging and sex. Reference cannot be made on transcranial Doppler (TCD), near infrared spectroscopy, or brain tissue oxygen monitoring (PbtO_2_) which were not included in this review because they are not utilized on a regular basis for bedside monitoring of TBI patients and are relegated for research-only measures at few specialized centers. Further, TCD and PbtO_2_ indices currently have no pre-clinical literature validating their ability to measure any aspect of the autoregulatory curve.

Sixth, PAx and RAC are not widely published across the literature because lots of works involving them are still exploratory. For this review, they had essentially no data. This stems from the new nature of these indices and the need for full-waveform ICP data to derive AMP through Fourier analysis techniques.^3,49^ Such expertise is not present at centers limiting the uptake of PAx and RAC for bedside monitoring at this time.

Seventh, the methods of quantifying age and biological sex in the selected studies could be considered crude and may not be sufficient in the assessment of their impact on CVR. Biological age, as estimated through emerging epigenetic techniques, may provide a quantified way in the future to determine age.^61,62^ Linking such data with high-frequency physiology in TBI patients may provide a superior assessment of the link between age and CVR. Biological sex, as measured through a binary designation (i.e., male vs. female), may be too crude to properly assess the link between sex and CVR^33,40,41,59^ given that there appears to be a discrepancy and intersectional nature between sex and age, regarding menopausal status.^33,40,41,59^

Yet another limitation has to do with the fact that the most aged patients, typically with varying overlaying comorbidities, are usually not included in most cerebral monitoring studies at most centers. This stems from specific medical and social discussions held during the admission process regarding patient comorbidities, degree of frailty, goals of care, patients' wishes/advance directives, and family/patients' views on aggressiveness of care. Thus, despite the interesting findings related to advanced age on ICP-based indices found in this review, there is the potential that this is underemphasized by the identified studies.

Finally, all the relevant studies were Addenbrooke/Cambridge hospital–based involving the same cohort of patients, except one study which involved 21 centers in the EU. This poses a potential issue given that this population is mainly Caucasian. Other potential issues involve the geographical location, genetics, and biological differences of the persons, which will vary to other populations (e.g., immigrants, Africans, etc.). Things to take into consideration are that treatment practices may vary in other parts of the world; for example, Asia, Africa, and South America do not follow BTF guidelines. Also, genetic differences among persons of different races could play a part in cerebrovascular response and host response to injury and advancing age.^34^

### Future directions

Going forward, to address these issues in a quest to improve neurotrauma care, numerous considerations need to be made. First, one solution for the issues of a small number of females, heterogeneity of patient populations, small study sizes, and use of the same cohort of patients is to conduct large, multi-center studies. This type of study is required to build power for male versus female assessment, harmonize data between centers, account for treatments received, build statistical power, and aid with adjusting for multiple comparisons. Second, more robust work into PAx and RAC need to be carried out in order to arrive at a place where they would eventually become validated for human use and contribute to neurotrauma care. This can be achieved by securing adequate funding, which would allow centers to then obtain the necessary equipment and hire the required experts to explore age, sex, PAx, and RAC using multi-center high-frequency physiological data sets. Third, in order to assess biological age, the required expertise is needed to carry out epigenetic research on large, multi-center TBI cohorts and obtain biological samples. With this, studies can then be carried out independently and combined to provide a superior assessment of the link between true biological age and CVR. Fourth, the study of biological sex needs to go beyond just the binary designation of male and female.

With all requirements in place, further subcategorization of females based on their menopausal status and hormonal profile is key to properly assess the link between sex, age, and CVR. Fifth, further to what was stated regarding age and sex, involvement of proteomic and genomic studies with adequate patient cohorts, relevant approvals, equipment, and expertise is key to then carry out age and sex subgroup studies in concert with insult burden measures (e.g., % time PRx >0, > 0.25, > 0.35, etc.). This will help unearth patterns and more in-depth, useful information.

A further consideration will be to carry out similar studies like the optimal cerebral perfusion pressure (CPPopt) Guided Therapy: Assessment of Target Effectiveness (COGiTATE) trial on a larger scale given that this trial confirmed the feasibility of CPPopt derivation, but percentage yield of CPPopt calculations was relatively low at ∼70%, and the study was not powered to detect differences in clinical outcome.^63^ However, previous literature on CPPopt highlights the strong association with this index and ICP-based cerebrovascular reactivity insult burden with 6-month outcome in TBI patients.^12,63–65^ Therefore, mastery of real-time signal artifact management, optimal curve fitting, and algorithmic derivation of CPPopt need to be achieved in order to improve yield before larger phase III trials are conducted.^12,63–65^ This will provide clarity on whether there exists a true impact of CVR-based therapeutic targets on outcome in this population.^12,63–65^

Also, the work into fully understanding how age and sex affect autoregulation post-TBI needs to be conducted by research into the local and systemic biochemical and inflammatory response to TBI.^34^ Finally, more diverse populations need to be assessed given that their location, environment, treatment protocols, hormone profiles, genome/proteome/epigenetic profiles, and healthcare budgets impacting care delivery are all key aspects to study.

## Conclusion

From the few studies identified, it was noted that with advancing age, PRx (with PAx and RAC to a lesser extent) becomes increasingly impaired. Further, preliminary data suggest that, potentially, females suffered worse CVR, compared to their male counterparts, during the acute phase of their ICU stay. However, all studies suffered from significant limitations, including small heterogeneous populations, small number of female patients, and grand averaging of large epochs of cerebral physiological data. Future multi-center studies on the impact of age and biological sex on continuously assessed cerebrovascular physiology are required, given that there still exists a substantial knowledge gap.

## Supplementary Material

Supplemental data

Supplemental data

## Supplementary Material

Supplementary Appendix SA

Supplementary Appendix SB

## Abbreviations Used

ICP: pulse amplitude of ICP
BTF: brain trauma foundation
CBF: cerebral blood flow
CPP: cerebral perfusion pressure
CPP_OPT_: optimal CPP
CVR: cerebrovascular reactivity
EU: European Union
GCS: Glasgow Coma Scale
ICP: intracranial pressure
ICU: intensive care unit
LLA: lower limit of autoregulation
MAP: mean arterial pressue
PAx: pulse-amplitude index
PRISMA: Preferred Reporting Items for Systematic Reviews and Meta-Analyses
PRx: pressure reactivity index
SD: standard deviation
TBI: traumatic brain injury
TCD: transcranial Doppler
