# Supplementary material for: Intracranial Pressure–Derived Cerebrovascular Reactivity Indices, Chronological Age, and Biological Sex in Traumatic Brain Injury: A Scoping Review
Source: Neurotrauma Rep. 2022 Jan 25;3(1):44–56. doi: 10.1089/neur.2021.0054 (PMC8804238; doi:10.1089/neur.2021.0054)
Supplement: Supplemental data [file Suppl_AppendixSB.docx]

**Appendix B: Sample MEDLINE Search Strategy**

1. traumatic brain injury.mp.
2. TBI.mp.
3. brain injury.mp.
4. head injury.mp.
5. head trauma.mp.
6. cerebral trauma.mp.
7. brain trauma.mp.
8. cerebral injury.mp.
9. concussion.mp.
10. neurotrauma.mp.
11. diffuse axonal injury.mp.
12. craniocerebral trauma.mp.
13. closed brain injury.mp.
14. acute brain injury.mp.
15. acquired brain injury.mp.
16. 1 or 2 or 3 or 4 or 5 or 6 or 7 or 8 or 9 or 10 or 11 or 12 or 13 or 14 or 15
17. pressure reactivity index.mp.
18. PRx.mp.
19. pulse amplitude of intracranial pressure.mp.
20. pulse amplitude.mp.
21. PAx.mp.
22. RAC.mp.
23. 17 or 18 or 19 or 20 or 21 or 22
24. 16 and 23

Mp = title, abstract, original title, name of substance word, subject heading word, floating sub-heading word, keyword heading word, organism supplementary concept word, protocol supplementary concept word, rare disease supplementary concept word, unique identifier, synonyms
